# The Role of Epigenetics in Congenital Heart Disease

**DOI:** 10.3390/genes12030390

**Published:** 2021-03-09

**Authors:** Tingsen Benson Lim, Sik Yin Roger Foo, Ching Kit Chen

**Affiliations:** 1 Department of Paediatrics, Yong Loo Lin School of Medicine, National University of Singapore, Singapore 119228, Singapore; tingsenlim@gmail.com; 2 Department of Medicine, Yong Loo Lin School of Medicine, National University of Singapore, Singapore 119228, Singapore; roger.foo@nus.edu.sg; 3 Genome Institute of Singapore, Agency for Science, Technology and Research, Singapore 138672, Singapore; 4 Division of Cardiology, Department of Paediatrics, Khoo Teck Puat-National University Children’s Medical Institute, National University Health System, Singapore 119228, Singapore

**Keywords:** cardiogenesis, cardiac development, epigenetics, transcriptional regulation, congenital heart disease

## Abstract

Congenital heart disease (CHD) is the most common birth defect among newborns worldwide and contributes to significant infant morbidity and mortality. Owing to major advances in medical and surgical management, as well as improved prenatal diagnosis, the outcomes for these children with CHD have improved tremendously so much so that there are now more adults living with CHD than children. Advances in genomic technologies have discovered the genetic causes of a significant fraction of CHD, while at the same time pointing to remarkable complexity in CHD genetics. For this reason, the complex process of cardiogenesis, which is governed by multiple interlinked and dose-dependent pathways, is a well investigated process. In addition to the sequence of the genome, the contribution of epigenetics to cardiogenesis is increasingly recognized. Significant progress has been made dissecting the epigenome of the heart and identified associations with cardiovascular diseases. The role of epigenetic regulation in cardiac development/cardiogenesis, using tissue and animal models, has been well reviewed. Here, we curate the current literature based on studies in humans, which have revealed associated and/or causative epigenetic factors implicated in CHD. We sought to summarize the current knowledge on the functional role of epigenetics in cardiogenesis as well as in distinct CHDs, with an aim to provide scientists and clinicians an overview of the abnormal cardiogenic pathways affected by epigenetic mechanisms, for a better understanding of their impact on the developing fetal heart, particularly for readers interested in CHD research.

## 1. Introduction

Congenital heart disease (CHD) refers to a heterogeneous collection of structural abnormalities of the heart or the great vessels present at birth. It is the most common birth defect in newborns, affecting >1 million live births per annum globally and causing 10% of stillbirths. The moderate and severe forms of CHD affect approximately 6–20 per thousand live-births [1], and is a major cause of infant mortality and morbidity in the developed world [2], accounting for ~40% of infant deaths in North America [3]. In the most recent era, the survival and outcome of CHD patients, including children with complex cardiac defects, have improved significantly such that more than 75% of CHD children who survive the first year may be expected to reach adulthood in developed countries [4]. This increased survival is due to advances in prenatal and postnatal diagnosis, innovation in surgical techniques, as well as improved clinical surveillance and translational research, which have dramatically improved the clinical management of CHD. As a result, there are more adults living with CHD than there are children with CHD [5], Nevertheless, the clinical course of children with complex CHD may be associated with many late sequelae, and affected children sometimes require subsequent invasive procedures and eventual heart transplantation due to heart failure caused by progressive ventricular dysfunction [6].

Thus, a greater understanding of the etiology of CHD is fundamental, in our effort to improve diagnosis, clinical management and counselling on risk of recurrence during subsequent pregnancies. With only 20–30% of cases traced to a known cause [7], the etiologies of CHD may be divided into genetic and non-genetic categories. While non-genetic etiologies of CHD such as environmental teratogens and infectious agents are widely studied [2], there is still a lot yet to be uncovered for the genetics and epigenetics of CHD [2]. With advancement of sequencing techniques, there has been greater appreciation of the significant genetic contribution to CHD in the recent 10–15 years, although its genetic basis was first recognized more than 3 decades earlier [8]. Epidemiology points to a strong genetic contribution in a proportion of CHD [9]. A greater concordance of CHD exists for monozygotic than dizygotic twins, and there is increased risk of recurrence among siblings or subsequent pregnancies [9]. Conversely, it is also notable that for a large proportion of CHD, particularly the severe forms, there is no other family history of CHD. This underlies a significant contribution from *de novo* genetic events [9]. Despite this, a specific causal genetic mutation is still only recognized in a minority of cases of sporadic CHD [10]. Currently, approximately 35% of CHD cases, with or without extracardiac malformations, can be attributed to genetic factors (Table 1) including monogenic (3–5%) or chromosomal (8–10%) anomalies, and copy number variants (3–25%). Environmental causes (2%) such as maternal diabetes, smoking or alcohol use, are recognized in ~20–30% of patients [1,11,12,13,14]. The genetics of CHD is also complex; a single candidate gene or genetic variant can produce a spectrum of heart malformations and may even occur in phenotypically-normal humans. Variation in genetic penetrance also occurs in affected families, resulting in a range of CHD phenotypes in the same pedigree [15,16].

There remains much to be learned about the genetic and epigenetic bases of CHD, which we are only now beginning to understand through tissue, animal and computational modelling. Multiple experimental animal models have provided invaluable understanding into the molecular mechanisms of CHD. In humans, the basis of these molecular mechanisms remains poorly understood owing to its complexity. Moreover, most CHDs are not part of any genetic syndrome i.e., sporadic malformations, with neither a family history nor a clear Mendelian inheritance of the disease [8]. Whole exome and genome sequencing efforts reveal mutations in genes that contribute to CHD [17], but mutations in protein coding genes only account for 35% of CHD [9]. Importantly, many of these protein coding genes are now recognized as those encoding components of the epigenetic molecular pathways (e.g., histone modifying enzymes), underpinning a causality hypothesis for “molecular epigenetics”, or pathways that regulate networks of gene expression [9].

The role of epigenetic regulation in cardiac development/cardiogenesis, using tissue and animal models, has been well reviewed elsewhere. Here, we summarize the current literature based on studies in humans, which have revealed associated and/or causative epigenetic factors implicated in CHD. We aim to provide scientists and clinicians an overview of the abnormal cardiogenic pathways affected by epigenetic mechanisms, for a better understanding of their impact on the developing fetal heart, particularly for readers interested in CHD research.

## 2. Overview of Congenital Heart Disease

As CHD comprises a heterogeneous group of cardiac malformations, the key clinical manifestation is dependent on the type of CHD. Since Maude Abbott developed The Atlas of Congenital Cardiac Disease more than a century ago, the classification of CHD has evolved substantially [18]. Broadly speaking, CHD can be classified, based on morphology and hemodynamics, into cyanotic and acyanotic CHD. Cyanosis is a bluish discoloration of the skin and mucous membrane resulting from reduced oxygen saturation in the circulating blood. Patients with cyanotic CHD have mixing of deoxygenated with oxygenated blood, with overall reduction of circulating oxyhemoglobin.

Common examples of acyanotic CHD include atrial septal defect (ASD) and ventricular septal defect (VSD). In ASD, there is a defect/hole in the septum/wall that divides the left and right atria; in VSD, the defect is in the septum between the left and right ventricles. Notably, VSD is one of the commonest congenital malformation of the heart. Atrioventricular septal defect (AVSD), also known as endocardial cushion or atrioventricular canal defect, comprises of a defect in atrioventricular septum, and malformed atrioventricular valves. In these defects, oxygenated blood shunts from the oxygen-rich (left) chambers to the oxygen-poor (right) chambers; therefore, these lesions are also termed left-to-right shunts. As the mixing is from the oxygenated to the deoxygenated chambers, there is no reduction in the oxygen saturation in the systemic circulation. However, this may result in increased pulmonary blood flow with consequent lung congestion.

Cyanotic CHD encompass defects such as tetralogy of Fallot, pulmonary atresia, transposition of the great arteries, double outlet right ventricle, persistent truncus arteriosus, tricuspid atresia, and total anomalous pulmonary venous drainage, to name a few. Table 2 summarizes the common types of cyanotic CHD.

TOF, TGA, DORV, and persistent truncus arteriosus are collectively known as conotruncal defects, as these lesions involve the conus and truncus arteriosus of the embryonic heart.

## 3. Overview of Cardiogenesis and Transcriptional Events during Cardiac Development

**Figure 1:**
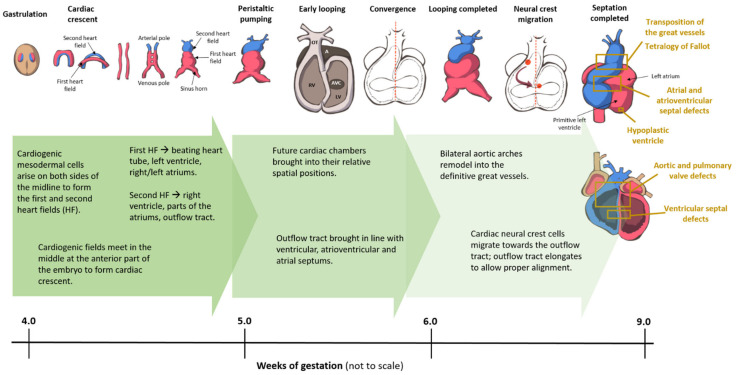
Cardiac development in the human embryo. This schematic shows the embryonic development of the human heart through first and second heart field formation, heart tube formation and pumping, looping, neural crest migration and septation, resulting in a fully developed heart at the end of gestation. HF: heart field.

**Figure 2:**
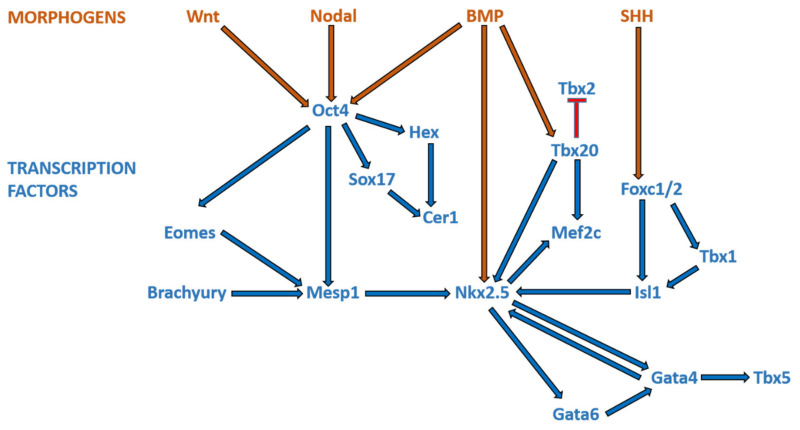
Morphogens and transcriptional factors regulating cardiac cell lineage and differentiation. *Note:* Blue arrows indicate activation; red arrows indicate inhibition.

Broadly speaking, CHD can be considered as the consequence of normal cardiac development gone awry. Heart development or cardiogenesis, is a complex developmental process (Figure 1) governed by multiple interlinked and dose-dependent pathways [19]. Owing to the complexity of the developmental processes governing morphogenesis of the heart, impairment in any of these steps understandably leads to CHD. These events increase the difficulty of identifying and characterizing the genetic risk factors for CHD, and emphasizes the importance of understanding cardiogenesis at the molecular level. Therefore, an overview of the cardiac developmental events and their transcriptional regulation (Figure 2) would be pertinent to provide context to understanding the developmental defects causing CHD.

### 3.1. Formation of the Linear Heart Tube

The earliest steps in cardiogenesis begin during gastrulation, which is the formation of the three germ layers—ectoderm, mesoderm, and endoderm; cardiomyocytes are derived from the mesoderm. At the site of gastrulation, canonical *Wnt* signaling, mediated by β-catenin, blocks differentiation of the mesodermal cells. As some mesodermal cells migrate toward the anterolateral border of the embryonic disc, they leave the *Wnt* expression domain and enter a domain of active *Wnt* inhibition; these cells make up the cardiogenic fields. The commitment of mesodermal precursors to cardiac progenitor cells involves signaling pathways acting in a coordinated fashion with a complex network of cardiac transcription factors. As pre-cardiac progenitors migrate towards the anterolateral aspect of the mesodermal layer, SMARCD3, a subunit of the SWI/SNF (switch/sucrose non-fermentable) chromatin remodeling complex, is expressed [20]. SMARCD3 allows GATA4, a zinc-finger transcription factor, to bind the enhancer regions of several transcription factors that initiate the cardiac gene program [21], including ISL1, NKX2-5, T-box 5 (TBX5), and myocyte enhancer factor 2c (MEF2C) [20,22]. Yin yang 1 (YY1), along with GATA4, binds directly to an enhancer region of NKX2-5 and activates early cardiac transcriptional activities [23].

The cardiogenic fields on either side of the midline consist of mesodermal cells, which now have the capacity of entering the cardiac lineage to differentiate into cardiomyocytes. This cardiac cell fate decision is coordinated by bone morphogenetic protein (BMP) growth factors produced by the ectodermal and endodermal cells at the lateral aspect of the embryonic disc. The cardiogenic fields then meet in the middle at the anterior part of the embryo to form the horseshoe-shaped cardiac crescent, and start expressing cardiac-specific transcription factors, namely Islet1 (Isl1) and Nkx2.5. The medial expansion of the cardiac crescent is regulated by BMP inhibitors expressed by the neural tube, and the posterior border determined by FGF growth factors secreted by the endoderm [24,25,26,27]. The two sides of the cardiac crescent subsequently fuse along the midline to form the primitive heart tube. The primitive heart tube may be subdivided into regions along the caudal-to-cranial axis: sinus venosus, primitive atriums, primitive ventricle, bulbus cordis (conus), and truncus arteriosus. The primitive heart tube starts to demonstrate peristaltic-like contractions, which are initiated at the venous pole, at approximately 5 weeks’ gestation [28,29,30].

### 3.2. Cardiac Looping

The primitive heart tube further develops by looping, a process in which it folds and twists. The mechanism that govern this process remains elusive; the heart tube loops through differential ballooning out of the chambers to acquire a C shape, and subsequently an S shape with ongoing development [31,32]. Normally, the primitive heart tube loops to the right with resultant D-looped heart. In some cases of CHD, the primitive heart tube loops anomalously to the left (L-looped). During looping, the heart tube lengthens through the continuous addition of newly differentiated cardiomyocytes, instead of proliferation of the cardiomyocytes of the heart tube [33,34]. Rapidly proliferating mesodermal cells distal to the venous pole provide these newly-differentiated cardiomyocytes. The high proliferative rate of these cardiac progenitor cells is mediated through canonical Wnt/β-catenin signaling [35,36]. These highly proliferative cardiac progenitor cells constitute what is often known as the second heart field (SHF), as opposed to first heart field (FHF) from which the initial heart tube is formed [37]. The FHF gives rise to the left ventricular free wall, part of the ventricular septum, and a portion of the atriums, whereas the SHF gives rise to the right ventricle, a portion of the ventricular septum, the outflow tracts, and part of the atriums [38].

The differentiation of progenitor cells into SHF or FHF lineages is orchestrated by differential signaling. BMP2, BMP4 [39], FGF8 [40], and non-canonical WNT [41] signals from the underlying endoderm promote the differentiation of the FHF progenitor cells. Once differentiated into cardiomyocytes, the cells cease proliferating, coinciding with the down-regulation of Isl1 and up-regulation of Nkx2.5 [37,42]. On the other hand, proliferation and multi-lineage differentiation of SHF progenitors is mediated by FGFs [43], sonic hedgehog (SHH) [44], and canonical WNT/β-catenin [45] signals. As they are being added to the heart tube, these SHF progenitors express the transcription factor Isl1. The segregation of the SHF cells to the inflow and outflow poles of the heart is regulated by the transcription factor Tbx1 [46,47]. From a proliferative state, SHF progenitors commit towards cardiac specification in the setting of increased BMP expression, as they migrate to the outflow tract [48,49]. BMP signaling promote cardiomyocyte differentiation by mediating the induction of *NKX2-5*, *MEF2C, SRF*, and *GATA4* expression [50,51]. In addition, BMP also upregulates *TBX2* and *TBX3*, which are required for reducing proliferation of myocardium within the outflow tract, atrioventricular canal, and sinus horns [52,53]. SHF cells have delayed commitment to the cardiomyocyte lineage compared with FHF cells. In this context, canonical WNT/β-catenin signaling is crucial for maintaining SHF progenitors in a proliferative state while inhibiting differentiation toward terminal lineages [54,55,56]. Conversely, non-canonical WNT signaling plays a critical role in cardiomyocyte differentiation [36]. WNT5A and WNT11, two non-canonical WNT ligands, are crucial for heart development [41,57], and they are thought to act via the suppressive effect of non-canonical WNT signaling on the canonical WNT/β-catenin pathway [58].

### 3.3. Septation

In mammals, the pulmonary and systemic circulations are separate, and these circulations are arranged in series with one another in adults. The physical separation of the initial single bloodstream into a systemic and pulmonary stream is achieved through septation, a process that divides the atriums, the atrioventricular (AV) valves, the ventricles, and the outflow tract during development. It is noteworthy that half of CHD involve septational abnormalities.

#### 3.3.1. Atrial and Atrioventricular Canal Septation

During ventricular development and as the cardiac tube lengthens, the atriums begin to differentiate at the inflow pole [59]. Although the atriums are formed symmetrically, the left and right atriums are morphologically distinct. The left-sided identity is mediated by the transcription factor Pitx2c. Consequently, the absence of Pitx2c expression results solely in right atrial identity and thus two morphological right atriums, referred to as right isomerism. Conversely, left isomerism ensues when there is ectopic expression of Pitx2c on the right side, resulting in two morphologically left atriums [60].

Although the atriums are the first structures to begin septation, they are the last to finish, with the foramen ovale normally remaining patent until after birth. The pulmonary venous confluence evaginates into the roof of the embryonic atrium at the beginning of the sixth week of gestation. Rostral to this, the crescent-shaped primary atrial septum grows from the dorsal wall of the atrium towards the AV canal. The leading edge of the primary atrial septum is covered by a mesenchymal cap. Anteriorly, this cap is contiguous with the anterior AV cushion, and posteriorly, with the dorsal mesenchymal protrusion (DMP) and the posterior AV cushion. Septation of the atrial chambers is achieved by the primary atrial septum as well as the endocardium-derived mesenchymal cap and AV cushions and SHF-derived DMP [61,62]. This septation process also divides the AV valve into the separate tricuspid and mitral valves. Consequently, defective DMP formation may lead to the formation of a common AV canal or complete AV septal defect in the most severe cases, and to an atrial septal defect in milder cases.

#### 3.3.2. Ventricular Septation

Following normal looping (D-looping), the primitive right and left ventricles end up relatively rightward and leftward to each other. However, in the anatomical position, the right ventricle is oriented anterior to the left ventricle. As the blood flows into the left ventricle, it crosses the bulboventricular foramen to the right ventricle and out through the unseptated outflow tract. As development progresses, inflow becomes more balanced and directed towards both ventricles, failing which a double inlet left ventricle [DILV] ensues. Ventricular septation occurs in the fourth week of gestation as the ventricular septum grows toward the AV canal and outflow tract from the apical and inferior portion of the junction between the primitive right and left ventricle. This forms the muscular part of the interventricular septum. The ventricular septum lengthens by apposition, a process during which cells are added to the septum at its base [63]. When apposition is aberrant, it can result in a hole or multiple holes in the ventricular septum, termed muscular ventricular septal defects (VSD). Septation of the ventricle is complete when the muscular septum meets the inlet (AV canal) septum between the tricuspid and mitral valves, and the outlet (conal or outflow tract) septum just below the now separate outflow tracts. If the inlet septum is not formed properly, this results in an AV canal type VSD. Similarly, if the outlet septum forms too far anterior or posterior (or deviated anteriorly or posteriorly), the muscular septum may not meet the outlet septum, causing a septal malalignment defect [64]. In the area at which these components of the ventricular septum meet, there is the thinner membranous septum.

#### 3.3.3. Outflow Tract Septation

The outflow tract is the part of the heart tube between the developing ventricles and the aortic sac, which is embedded in the pharyngeal arches and is connected to three symmetrically paired pharyngeal arch arteries. Septation of the outflow tract starts distally in the sixth week of development, and proceeds proximally, to its completion a week later [65]. At the end of the eighth week of gestation, the pharyngeal arch arteries remodel into the arterial pole of the heart, resembling the postnatal configuration [66].

Initially, the outflow tract increases in length by the addition of SHF-derived newly differentiated cardiomyocytes [65,67,68,69], and by migration of the cardiac neural crest cells [42,70]. Cardiac neural crest cells are a population of cells that are crucial to the separation of the pulmonary and systemic circulations. They migrate from the dorsal neural tube, and surround the developing pharyngeal arch arteries whilst remodeling the arch. Two prongs of neural crest cells continue to migrate towards the outflow tract on each side of the truncus. The migration of the neural crest cells into the heart is a complex process that is regulated by Bmp, Wnt, Fgf, and Semaphorin signaling [71]. The outflow tract cushions lay in a spiraling fashion, which may contribute to the apparent spiraling configuration of the aorta and pulmonary artery in the fully developed heart [65,72].

When the outflow tract cushions do not fuse over their entire length, a persistent truncus arteriosus or common arterial trunk ensues. When the fusion defect is limited to the proximal outflow tract cushions, this results in a juxta-arterial or outlet VSD [73,74]. Failure of the rotation of the outflow tract results transposition of the great arteries and double-outlet right ventricle. Because cardiac neural crest provides cells to the outflow tract, and these cells secrete growth factors that are critical for normal development of the surrounding tissue, interference with the neural crest results in a spectrum of abnormalities including a persistent truncus arteriosus, pulmonary atresia and double outlet right ventricle [71,75]. Indeed, disruption of the gene *Tbx1* leads to defects in neural crest migration in DiGeorge syndrome with conotruncal defects [47].

## 4. Epigenetics and Congenital Heart Disease

The expression of thousands of genes in millions of cells in a single organism must be tightly regulated, during development and throughout its life. Although the genotype of most cells of a given organism is the same (except the gametes and the cells of the immune system), multiple cell types and functions emerge owing to highly regulated gene activity. Gene activity is largely mediated by transcriptional regulation, which is in turn orchestrated by epigenetic mechanisms. Although the definition of epigenetics may have evolved over time, Conrad Waddington in the 1950s proposed that, *“An epigenetic trait is a stably heritable phenotype resulting from changes in a chromosome without alterations in the DNA sequence”* [76]; and this can involve the heritability of a phenotype, passed on through either mitosis or meiosis. The mechanisms that govern epigenetics comprise of DNA methylation, histone modifications, higher-order chromatin structure, and the activity of certain non-coding RNA species [77]. Overall, epigenetics are heritable, and mutations in single gene responsible for these mechanisms can lead to dysregulation on an array of genes leading to polygenic diseases including CHD. Here, we describe the list of genes implicated in CHD across the different mechanisms. Table 3, Table 4 and Table 5 summarize the current available literature of human studies.

### 4.1. DNA Methylation

The first epigenetic mechanism discovered, DNA methylation is involved in a variety of biological process including embryonic development, X-chromosome inactivation and genomic imprinting [112]. DNA methylation refers to the covalent addition of a methyl group at the 5’ position of the cytosine nucleotide. In the mammalian genome, methylation is restricted to CpG dinucleotides, which are largely depleted from the genome except at short genomic regions called CpG islands, which commonly reside at promoter regions. CpG islands are 300–3000 base pair regions that tend to be non-methylated [113]. DNA methylation is catalysed by DNA methyltransferases (DNMTs), including DNMT1, DNMT3A, and DNMT3B. DNMT3A and DNMT3B are known to be responsible for *de novo* methylation, and are essential for embryonic development [114]. Maintenance of methylation, i.e., the transfer of methylation patterns to daughter cells is carried out by DNMT1, and DNMT1 disruption results in loss of cell proliferation and cell death [115]. On the other hand, demethylation is achieved by the activity of ten-eleven-translocases (TET) enzymes. TET1 drives DNA demethylation by converting 5-methylcytosine to the intermediate 5-hydroxymethylcytosine [116].

Approximately 70% of gene promoters contain CpG islands, and methylation of these areas results in modification of gene expression patterns [117]. Before cardiomyocyte maturation during embryonic life, cardiomyocyte development is accompanied by a change in DNA methylation status, and the DNA methylome consisted of a demethylation wave running through cardiac gene bodies, including genes encoding sarcomeric proteins, followed by *de novo* DNA methylation after birth [118]. Thus, aberrant DNA methylation through its transcriptional consequences may have an important role in CHD (Table 3). Since folate is a key source of the one carbon group used to methylate DNA [119], the role of methylation abnormalities in CHD has been explored by studying the methylation regulatory folate-pathway [120,121]. Furthermore, maternal folate supplementation has been reported to decrease the risk of CHD associated with Down syndrome (trisomy 21) [122].

#### DNA Methylation and CHD 

In a Dutch population-based case-control study of 143 children with CHD and 186 healthy children, CHD children had increased concentrations of biomarkers of methylation, and high concentrations of methylation biomarkers in very young children are associated with complex CHD [123]. In their subgroup analysis, Down syndrome (DS) with CHD was associated with a global hypomethylation status [123]; this was similarly reported in mothers of children with Down syndrome [124], suggesting the heritability of the global methylation state and a possible role in the segregation of chromosomes. Genome-wide DNA methylation analysis performed on DNA from 60 newborns with various CHDs and 32 controls showed significant differences in methylation patterns in hundreds of genes in newborns with different types of CHD; this study illustrated the potential for development of accurate genetic biomarkers for CHD detection in newborns [125], or even fetuses.

Methylenetetrahydrofolate reductase (MTHFR) is one of the major enzymes of the folate metabolism pathway. Decrease in folate metabolism can lead to DNA strand breakage and abnormal segregation of chromosomes [126,127]. To compare the levels of *MTHFR* promoter methylation, three groups comprising of mothers of DS without CHD, mothers of DS with CHD, and age-matched control mothers, were evaluated [89]. The study demonstrated a significant increase in methylated promoter region of *MTHFR* gene in mothers of DS with CHD compared to other groups, highlighting the association of *MTHFR* promoter hypermethylation in mothers of DS with cardiac defects [89]. In a global DNA methylation comparing fetal heart tissue from DS with CHD, DS without CHD, isolated CHD, and controls, hypermethylation of the *GATA4* gene body was present in fetuses with DS with or without CHD, as well as in fetuses with isolated heart malformations [84]. Moreover, hypermethylation of several intragenic sites at the *MSX1* gene, involved in outflow tract morphogenesis, was found in a fetus with isolated CHD, suggesting these epimutations likely contribute to the pathogenesis of the malformation by *cis*-acting effects on gene expression [84]. Using microarrays dedicated for assessment of gene promoter methylation and whole genome expression profiles between two groups of DS patients, with and without CHD of endocardial cushion-type (e.g., atrioventricular septal defect), significant hypermethylation of the *NRG1* gene promoter region is observed in the group of children with CHD [93].

To understand epigenetic mechanisms that may play a role in the development of VSD, genome-wide DNA methylation assay on placentas of term babies with isolated VSD and unaffected controls showed significant differential methylation in the placental DNA of VSD cases compared to controls [128]. Although not validated, the biological processes and functions for many of these differentially methylated genes were previously known to be associated with heart development, including ventricular development (*HEY2*, *ISL1*), looping (*SRF*), cardiomyocyte differentiation (*ACTC1*, *HEY2*), septal development (*ISL1*), and Notch signaling pathway (*HEY2*, *HEYL*). A global analysis of DNA promoter methylation from fetal myocardial tissue samples with VSD and normal fetal samples uncovered 70 and 85 genes that were regulated by hypermethylation and hypomethylation, respectively, in VSD [79]. Of these, the promoters of *KIAA0310*, *RAB43*, and *NDRG2* were validated to be hypermethylated, while *SIVA1* was validated to be hypomethylated [79]. Hypermethylation of the *NOX5* promoter was more common in VSD fetuses than in normal fetuses and this was corroborated with a decrease in *NOX5* expression with increased *NOX5* promoter methylation, suggesting that hypermethylation of the *NOX5* gene may be involved in the pathogenesis of VSD [78]. *NOX5* is part of the NADPH oxidase (NOX) family and is involved in cell proliferation, transformation, differentiation and apoptosis [129,130,131]. In an epigenome-wide DNA methylation study of 84 children with perimembranous VSD (pmVSD) and 196 controls, differential methylation of a CpG locus (cg17001566) within the *PRDM16* gene was identified, suggesting an association with pmVSD pathogenesis [132]. *PRDM16* functions as a repressor of TGF-β signaling, controlling tissue morphogenesis crucial during cardiogenesis. Animal models and studies using human cardiac tissue have shown that disruptions in *PRDM16* function affect cardiac development [133,134].

The long interspersed nucleotide element-1 (LINE-1) is a repetitive element that constitutes 17–25% of the human genome [135]. Methylation levels of LINE-1 are representative of genome-wide methylation status and play an important role in maintaining genomic stability and gene expression. Hypomethylation of LINE-1 was shown to be associated with increased risk of TOF [80]. In agreement with this observation, a similar study on TOF patients and controls also showed lower global DNA methylation levels including LINE-1 [81]. Genome-wide methylation assay in newborn blood in 24 non-syndromic TOF cases and 24 unaffected matched controls identified 64 significantly differentially methylated CpG sites in TOF cases, of which 25 CpG sites had high predictive accuracy for TOF which warrant further validations and investigations [136]. *TBX20* is expressed in the precursor structure of the cardiac valves and atrioventricular septa [137,138,139]. The methylation level of *TBX20* promoter regions were generally lower in patients with TOF compared to controls with an inverse expression level of *TBX20* transcripts in the right ventricular outflow tract myocardium, suggesting a role for *TBX20* in the pathogenesis of TOF [90,91]. *NKX2.5* and *HAND1*, encode transcription factors that regulate specific phases of heart development [140]. Promoter methylation analysis of these genes revealed certain regions in *NKX2.5* and *HAND1* had significantly higher median methylation level in TOF patients compared to controls [82]. Expression levels of these genes correlated with their promoter methylation status suggesting its association with regulation of gene transcription in TOF patients and may play an important role in the pathogenesis of TOF [82]. The same group performed a similar analysis on the methylation status on the promoter regions of 71 CHD candidate genes in TOF and control specimens [83]. Of these, the methylation values of *EGFR*, *EVC2*, *TBX5* and *CFC1B* were significantly correlated with their mRNA levels, suggesting that aberrant promoter methylation of these CHD candidate genes may contribute to the TOF development [83]. *ZFPM2* encodes a zinc-finger protein that interacts with *GATA4* to modulate its activity and is required for normal cardiogenesis [141]. Methylation levels in the CpG island shore of *ZFPM2* promoter and mRNA expression levels were significantly higher and lower, respectively, in patients with TOF compared to control, suggesting aberrant methylation status of *ZFPM2* may be associated with its gene transcription regulation in the TOF patients [86]. The human *p16^INK4a^* gene is a tumor suppressor gene and conditional *p16* knockout mouse models developed heart defects [142,143]. The methylation of CpG islands in *p16^INK4a^* was significantly higher, with reduced transcript and protein levels, in TOF patients compared to controls [87].

By comparing genome-wide DNA methylation with genome-wide expression data from myocardial biopsies of TOF, VSD, and control patients, hypermethylation of CpG islands located in the promoter of *SCO2* was identified in TOF and VSD patients [85]. This observation was corroborated with significant reduction at the transcriptional level of *SCO2* in TOF and VSD specimens [85]. These CpG may harbor binding sites of TFs playing a role in early cardiac development, as exemplified by the findings of myofibrillar disarray, narrowing of the heart tube and dilated cardiomyopathy increasing with age in cardiac-specific knockdown of *SCO1* and *SCO2* homologs in drosophila. *SCO2* encodes a cytochrome C oxidase assembly protein that plays a role in the aerobic production of ATP across the inner mitochondrial membrane [144].

Combining artificial intelligence and genome-wide DNA methylation analysis on isolated non-syndromic coarctation (CoA) cases and controls using neonatal blood spot revealed 65 different CpG sites located in 75 genes in CoA subjects [145]. Gene ontology analysis yielded epigenetic alterations in important cardiovascular developmental genes and biological processes [145]. Genome-wide DNA methylation analysis on neonatal dried blood spots identified significantly-altered CpG methylation at 59 sites in 52 genes in aortic valve stenosis (AVS; *n* = 24) subjects as compared to controls (*n* = 24) [146]. Although not experimentally verified the *APOA5* and *PCSK9* genes, known to be involved in AVS, have significant epigenetic changes [146]. Although monozygotic (MZ) twins share nearly all of their genetic variants before and after birth they can also be discordant for common complex diseases, such as type 1 [147] and 2 [148] diabetes, autism [149,150], and different types of cancer [151]. In a genome-wide sequencing and methylation analysis on whole blood from a MZ twin pair discordant for double outlet right ventricle (DORV), a total of 1566 differentially methylated regions (DMRs) between the MZ twins was uncovered [92]. Twenty percent of the DMRs were located within 2 kb upstream of transcription start sites [92]. Of note, *ZIC3* and *NR2F2*, both of which have roles in heart development [152,153], were found to have hypermethylated promoters in both the diseased twin and additional patients suffering from DORV [92]. Assessing the association of TGA with SNPs (single nucleotide polymorphisms) in *DNMT1* on 206 patients with complete TGA and 252 healthy children, there was suggestion that the the C/T genotype of the rs16999593 SNP in DNMT1 (compared to T/T genotype) might decrease the risk of complete TGA in the Southern Chinese population [154].

Interestingly, the use of placental tissue to identify methylation markers in CHD has recently been explored as a potentially useful surrogate for evaluating the development of heart tissue. One study found 165 significantly differentially methylated CpG loci in 8 non-chromosomal, non-syndromic TOF cases compared with 10 unaffected newborns, in 165 separate genes [155]. Methylation analysis on placentas of 8 term subjects with isolated VSD and 10 unaffected controls revealed 80 CpGs and eight miRNAs that had the potential to accurately detect VSD [128].

### 4.2. Histone Modifications and Chromatin Modeling

Nucleosome is a structural unit of a eukaryotic chromosome consisting of about 150 bp of DNA sequence wrapped around a core of histone proteins. Each histone, also known as a histone octamer, is composed of two units of the histone proteins H2A, H2B, H3, and H4, each. Importantly, H3 and H4 histones have tails protruding from the nucleosome that can be modified post-translationally to alter the histone’s interactions with DNA and nuclear proteins leading to epigenetic changes. These modifications primarily include methylation and acetylation, but can also include ubiquitination, phosphorylation, and sumoylation. Such alterations can effectively change chromatin conformation, hence, permitting the control of gene expression. Histone methyltransferases (HMT), demethylases, acetyltransferases (HAT), deacetylases (HDAC), and ATP-remodeling factors together comprise the chromatin remodeling complexes. Table 4 summarizes the current literature on the involvement of histone modifications in CHD.

#### 4.2.1. Histone Modifications and CHD 

HMT catalyze the transfer of methyl group(s) to lysine and arginine residues of histone proteins. In an effort to deduce the disease mechanism of non-syndromic patent ductus arteriosus (PDA), combine genome-wide linkage analysis and WES study identified independent missense mutations in *PRDM6* [102]. The authors showed *PRDM6* encodes a nuclear protein that is specific to vascular smooth muscle cells (VSMC), has HMT activities [156], and acts as a transcriptional suppressor of contractile proteins. In vitro studies showed *PRDM6* reduced dimethylation of H3K9, and increased dimethylation of H4K20, whereas variants of *PRDM6* (p.Arg549Gln and p.Cys263Ser) had completely opposite effects [102]. Histone H3 trimethylation at lysine 36 (H3K36me3) is a key gene transcription regulator epigenetic mark. The Wolf-Hirschhorn candidate protein 1 (WHSC1), an H3K36me3-specific HMT, is crucial in normal cardiogenesis [94]. The deletion of *WHSC1* results in the Wolf-Hirschhorn syndrome (WHS) [94,96], a microdeletion syndrome characterized by delayed growth and underdevelopment of several organs and CHD, including hypoplastic left heart syndrome (HLHS) [95].

Histone acetylation occurs by the enzymatic addition of an acetyl group (COCH_3_) from acetyl coenzyme A. The process of histone acetylation is tightly involved in the regulation of many cellular processes, and the modifying enzymes involved in histone acetylation are called histone acetyltransferases (HATs). HATs play a critical role in controlling histone H3 and H4 acetylation. A study involving parent-offspring trios whole exome sequencing (WES) on severe CHD cases and controls revealed a significant proportion of *de novo* mutations found in genes related to histone 3 lysine 4 (H3K4) methylation, or ubiquitination of H2BK120 which is required for H3K4 methylation, and in SMAD2 which regulates H3K27 [97].

As a NODAL pathway inhibitor [157], the *EBAF* (endometrial bleeding-associated factor, a.k.a. *LEFTY2*, left-right determination factor 2) plays a critical role during mammalian cardiac development [158,159]. Expression of *EBAF* in disease tissues of VSD patients was lower compared to healthy fetuses, and chromatin immunoprecipitation (ChIP) assays showed that HAT p300 is involved in the activation of *EBAF* through inducing hyperacetylation of histone H4 at the *EBAF* promoter [98].

*KAT2B* (lysine acetyltransferase 2B) is an important HAT epigenetic factor in the TGF-β signaling pathway and alteration in this gene has been associated with the etiology of cardiovascular diseases [160,161]. In a targeted sequencing study of 400 Chinese individuals for the *KAT2B* allele, it was uncovered that the variants rs3021408 and rs17006625 in the *KAT2B* gene were associated with the risk of CHDs in the Chinese Han population, suggesting the possible involvement of the *KAT2B* gene in the etiology of CHD [101]. Notably, the SNP rs3021408 is located in the homology domain and E3 ligase activity region, while rs17006625 is located between the homology and AT domains of the KAT2B protein [101].

*KANSL1* belongs to a HAT complex, and is involved in modifying the acetylation state of histone H4, mainly H4K16 [162,163]. The 22q11.2 deletion syndrome (22q11DS/DiGeorge syndrome/velo-cardio-facial syndrome) is caused by a heterozygous deletion of 1.5–3 Mb on chromosome region 22q11.2, and is the most common microdeletion disorder in humans with an incidence of 1 in 4000 live births [164,165]. About 60%-70% of the affected individuals have CHD, mostly of the conotruncal type (e.g., tetralogy of Fallot, transposition of great arteries, persistent truncus arteriosus) [166]. Analysis of genomic DNA from 253 patients with 22q11.2DS showed an association between CHD and a microduplication located in region 17q21.31 that includes multiple exons of *KANSL1* suggesting that *KANSL1* plays a role as a modifier gene in 22q11.2DS patients [103]. In a separate WES study on 89 individuals with 22q11DS and severe CHD, 20% of these had rare deleterious single-nucleotide variations (rdSNVs) in four genes including *JMJD1C*, *RREB1*, *MINA*, and *KDM7A*, all of which are involved in demethylation of histones (H3K9, H3K27) [100].

Histone ubiquitination differs substantially from the other modifications because of its large modification with the ubiquitin polypeptide. The dominant form of ubiquitinated histones are monoubiquitinated H2A (H2Aub) and H2B (H2Bub). H2Bub1 is catalyzed by the RNF20 complex consisting of RNF20, RNF40, and UBE2B [167]. A recent study identified three CHD patients with monoallelic mutations (RNF20 and UBE2B *de novo* and RNF40 inherited) affecting the RNF20 core complex [99]. H2Bub1 pathway modulation during development leads to abnormal cardiac left-right patterning [99].

#### 4.2.2. Chromatin-Remodeling Complexes and CHD

At a more complex level of epigenetic regulation, ATP-remodeling factors are involved, and there are four groups of SWI-like ATP-dependent chromatin-remodeling complexes: (1) the SWI/SNF (switching defective/sucrose nonfermenting) including the main ATPase subunit of the complex *Brg1*; (2) ISWI (imitation switch); (3) CHD, chromodomain helicase; and (4) INO80 (inositol requiring 80) complexes. These complexes play a crucial role in cardiac development during the early stages.

The SWI/SNF complex is comprised of 9–12 subunits. It is recruited to the promoters of numerous target genes by sequence-specific transcription factors; at the promoters, these complexes slide or evict nucleosomes near the transcription start site (TSS) to regulate RNA Polymerase II occupancy and therefore, transcriptional activity. Transcription can be upregulated or downregulated depending on whether a transcriptional activator or repressor recruits the SWI/SNF complexes. Each SWI/SNF complex contains DNA-dependent ATPase subunit encoded by either *BRM* (brahma, also known as *SMARCA2*) or *BRG1* (brahma-related gene 1, also known as *SMARCA4*) as alternative catalytic subunits [168]. The energy of ATP hydrolysis is utilized to disrupt histone-DNA contacts, and move nucleosomes away from the TSS or toward the TSS. The non-catalytic subunits of SWI/SNF are referred to as BAFs (BRG1 or BRM-associated factors, followed by a number to indicate the molecular mass of the protein). Each SWI/SNF complex contains a single ARID (AT-rich interacting domain)-containing subunit. SWI/SNF complexes may be classified into BAF and PBAF (polybromo-associated BAF) complexes based on their catalytic and ARID subunits. BAF complexes are catalyzed by either BRG1 or BRM and incorporate either BAF250a (a.k.a. ARID1a) or BAF250b (a.k.a. ARID1b), whereas PBAF complexes are exclusively catalyzed by BRG1 and incorporate BAF200 (a.k.a. ARID2).

SWI/SNF complexes play a crucial role in cardiomyocyte development [169,170,171]. *Brg1* interacts with *Tbx5* [171], and the *BAF60c* subunit facilitates a physical interaction between *brg1* and the cardiogenic transcription factors *NKX2-5, TBX4*, and *GATA4* [172]. Several exome sequencing projects have identified *de novo* mutations in four different SWI/SNF subunits in three genetic syndromes that include CHDs: (1) Coffin-Siris syndrome (CSS); (2) Nicolaides-Baraitser syndrome (NCBRS); and (3) ARID1B-related intellectual disability (ID) syndrome [173,174,175,176,177]. Patients with CSS, NCBRS, and ID syndromes exhibit various features including severe intellectual deficits, and CHD such as ASD, VSD, PDA, mitral and pulmonary atresia, mitral and tricuspid regurgitation, aortic stenosis, coarctation of the aorta, and single right ventricle [173]. Methylation profiling and gene expression studies in the myocardium of CHD patients and normal controls showed hypomethylation and decreased expression of *BRG1* [88].

Based on the discourse above, there is evidence that TBX5 is at a focal point of human CHD genes. However, the spatiotemporal nature of its expression and the context-specific manner of its regulation makes in vivo human studies challenging, if not impossible. In this regards, human in vitro systems have emerged to be powerful tools for gene-centered cardiac disease modeling at single-cell resolution. The controlled differentiation of induced pluripotent stem cells (iPSCs) [178] into cardiomyocytes provides a unique platform with which to establish personalized models of CHD that feature patient-specific factors and phenotypes. Using CRISPR-Cas9-mediated genome editing to target *TBX5*, a dose-sensitive requirement of TBX5 for human ventricular cardiomyocyte differentiation and function was demonstrated at single-cell resolution. The investigators used single-cell RNA-sequencing (scRNA-seq) across a time course of differentiation and discovered discrete gene expression responses to reduced TBX5 dosage in cardiomyocyte subpopulations. From these data, the investigators predicted genetic interaction between *TBX5* and *MEF2C*, manifesting as muscular VSD [179].

### 4.3. Non-Coding RNA

Non-coding RNAs (ncRNAs), as the name suggests, are RNA molecules that are not translated to proteins. ncRNAs involved primarily in the control of gene expression include long ncRNA (lncRNA) and microRNA (miRNA). LncRNAs, >200 nucleotides in length, are located in the nucleus or cytoplasm [180,181], and can directly interact with chromatin remodeling complexes to further modulate transcription [182]. Meanwhile, miRNAs are single-stranded RNAs of approximately 19–24 nucleotides that negatively affect gene expression at the post-transcriptional level, either by guiding mRNA degradation or by preventing protein translation [183,184]. Since the advancement of next generation sequencing technologies over the years, our understanding of ncRNAs in cardiac development has improved significantly. Despite increasing evidence on differentially expressed miRNAs in CHD patients, the functional contribution of individual miRNAs remains to be elucidated. Regardless, miRNAs and lncRNAs are attractive clinical biomarkers as they remain stable in blood and urine, and evade RNA degrading enzymes.

#### 4.3.1. The Roles of Non-Coding RNA in Cardiac Differentiation

miRNAs have been recognized as part of the regulatory network governing developmental or physiological processes, including cardiac differentiation and morphogenesis. In mice, cardiac precursor specific deletion of *Dicer*, an RNase critical for generation of mature miRNAs from pre-miRNAs, resulted in embryonic lethality due to dilated cardiomyopathy, ventricular hypoplasia, and heart failure. Although cardiomyocyte-specific deletion of *Dicer* resulted in a milder phenotype as mice survived to birth, they too died soon after from dilated cardiomyopathy and heart failure [185].

Several miRNAs which play important roles during cardiac development, are specifically expressed in the developing heart and skeletal muscles such as the miR-1 and miR-133 families, miR-1-1/miR-133a-2 and miR-1-2/miR-133a-1 [183,186]. In the developing heart, they are regulated by serum response factor (SRF) and MEF2 transcription factors, which are intricately involved in cardiomyocyte differentiation [187,188]. In animal models, homozygous deletion of miR-1-2 resulted in embryonic or perinatal mortality because of cardiac defects, including VSD, heart failure, and arrhythmia [189]. Overexpression of miR-1-2 in fetal cardiomyocytes, resulted in thinning of the ventricular wall and heart failure [189]. Mice lacking both miR-133a-1 and miR-133a-2 developed severe heart failure because of VSD and dilated cardiomyopathy [190]. Deletion of individual miR-1-1/miR-133a-2 or miR-1-2/miR-133a-1 gene clusters did not affect survival to birth, cardiac morphogenesis, or function, suggesting functional redundancy [191]. However, double knockout of both miR-1-1/miR-133a-2 and miR-1-2/miR-133a-1 caused embryonic lethality with marked thinning of the compact ventricular myocardium and impaired cardiomyocyte maturation and proliferation. Not surprisingly in humans, a study profiling whole transcriptome and analyzing the relationship of miRNAs and mRNAs of right ventricular tissues of a homogeneous group of 22 non-syndromic TOF patients, and compared to profiles obtained from right and left ventricular tissue of normal hearts, investigators identified a list of disease-related miRNA-mRNA pairs that comprised novel as well as known miRNAs which are essential to cardiac development, and these included miR-1 and miR-133 [192].

#### 4.3.2. Non-Coding RNA and CHD

##### MicroRNAs and TOF/Cyanotic CHD 

Table 5 summarizes the current literature on the involvement of miRNA in CHD. Dysregulation of miR-184 has been shown to be implicated in the process of cardiomyocyte hypertrophy [193,194]. miR-184 was significantly downregulated in patients with cyanotic CHD compared to controls. Moreover, in vitro studies showed inhibition of miR-184 markedly decreased cell viability and induced apoptosis under hypoxic conditions suggesting that miRNA-184 may contribute to the development of cyanotic CHD via decreasing proliferation and inducing apoptosis of cardiomyocytes [108].

Connexin 43 (Cx43) plays an important role in cardiac development by providing direct intercellular communication [195], and *Cx43* overexpression may participate in the pathogenesis of TOF [195,196]. In a study to investigate the expression of *Cx43* and its related miRNAs in patients with TOF, the expression of *Cx43* and ten *Cx43*-related miRNAs in the myocardium was significantly increased, and were inversely correlated with the expression of miR-1 and miR-206 in the TOF patients when compared to controls. This suggested that miR-1 and miR-206, which impair *Cx43* expression, might be involved in the pathogenesis of TOF [106].

Comparison of normal (*n* = 8) and TOF tissues (*n* = 15) yielded 32 and 875 differentially expressed miRNAs and genes, respectively [197]. Genes in the network were enriched into 14 function clusters with protein localization being the most significant one [197]. The described microRNA–mRNA genetic network included miR-124 and miR-138, which may play pivotal role in protein localization, and possibly contributing to TOF.

Further evidence of the functional role of microRNAs in TOF pathogenesis included the identification that miR-940 was highly expressed in the normal human RV outflow tract but dramatically downregulated in TOF patients. Functional analysis revealed that miR-940 downregulation blocked cardiac resident cell migration by targeting *JARID2*, a gene involved in cardiac outflow tract development [198].

Several studies have provided evidence of association between distinct miRNA single nucleotide variants and the occurrence of TOF. Although evidence of functional implications are lacking, large dataset has provided evidence of the involvement of multiple deregulated miRNAs in TOF. miR-196a is an upstream regulator of *Hoxb8* and *Sonic hedgehog* (*Shh*) in vivo in limb development [199], and was suggested to be involved in heart development and CHD due to *Shh* signaling requirement throughout cardiac septation to valve formation [61,200]. It was reported that a genetic variant of rs11614913 in the miR-196a2 sequence could alter mature miR-196a expression and target mRNA binding [201]. In a three-stage case-control study, in which the associations identified in the initial stage were independently evaluated in the subsequent stages, the genetic variant miR-196a2 rs11614913 CC (compared to CT or TT) was associated with a significantly increased risk of CHD in all three stages combined [104]. A genotype-phenotype correlation analysis using 29 cardiac tissue samples of CHD showed rs11614913 CC was associated with significantly increased mature miR-196a expression. In vitro binding assays further revealed that the rs11614913 variant affects HOXB8 binding to mature miR-196a [104]. This observation was consistent with another independent study of 173 Chinese patients with TOF and 207 non-CHD controls using whole blood samples [202]. On the other hand, a similar association study reported the miR-196a2 (rs11614913, T > C) homozygous CC variant and the C allele were associated with a significantly reduced risk of ASD [203]. Hence, the exact role of the SNP rs11614913, and the related molecular mechanism that occurs during ASD pathogenesis needs to be elucidated.

Additional evidence of association between distinct miRNA single nucleotide variants and occurrence of CHD included the link between SNP rs4705343 of miR-143/145 and conotruncal-type CHD in an association study on whole blood on 259 conotruncal-type CHD patients and 303 control subjects [204]. In a case-control study, the miR-138 rs139365823 minor allele A appeared to increase its own expression of miR-138, and enhanced the post-transcriptional repression of target genes, thus, potentially conferring a predisposition to cardiac malformations within the studied population of CHD and non-CHD cases [111]. Notably, disruption of miR-138 resulted in an abnormal cardiac morphology and function during zebrafish embryonic development by targeting the retinoic acid pathway [205]. Conversely, in a study correlating pri-miR-124 (rs531564) polymorphism and CHD susceptibility in 432 sporadic patients and 450 controls, gene expression and miRNA profiles suggested that the minor C allele of SNP rs531564 may be involved in the reduction of the risk of CHD in a way that interacts with the intrauterine hypoxic environmental factors [206].

In patients with HLHS, it was observed that a total of 93 miRNAs were differentially regulated in HLHS hearts, in a study comparing the miRNA profile of the RV in HLHS patients and those of non-failing hearts [207]. Of these, miR-137 and miR-145, which were downregulated in the RV of HLHS patients, directly regulate the expression of *FOG-2*, a transcription factor that modulates the expression of *GATA4*, *GATA5* and *GATA6*, essential players in cardiac development. miR-204 was upregulated in the RV of HLHS patients, similar to patients who suffer from hypertrophic cardiomyopathy. Other differentially regulated miRNAs included miR-208, miR-30 and miR-378; these miRNAs have been implicated in the fetal program during heart failure (miR-208) and in RV hypertrophy and failure (miR-30 and miR-378) in an animal model.

##### MicroRNAs and Septal Defects

Gene expression data analysis of DS patients without CHD, and DS patients with AVSD revealed the involvement of miR-518a, miR-518e, miR-518f, miR-528a and miR-96 in the pathogenesis of AVSD in DS patients, potentially by targeting *AUTS2* (autism susceptibility candidate 2) and *KIAA2022* (a.k.a. X-linked mental retardation protein) [110]. *AUTS2* contributes to the differentiation of human embryonic stem cells into cardiomyocytes [208], and a is involved in NOTCH signaling pathway [209]. *KIAA2022* plays a role in regulating cell-cell and cell-matrix adhesion and migration [210], and it has been shown that the dysregulated cell-cell and cell-matrix adhesion signaling pathways contribute to heart birth defects during heart organogenesis [211].

In a study to characterize circulating miRNA expression in patients with VSD, miRNA microarray analysis revealed seven miRNAs (let-7e-5p, miR-155-5p, miR-222-3p, miR-379-5p, miR-409-3p, miR-433, miR-487b) and one miRNA (hsa-miR-498) were downregulated and upregulated, respectively, in patients with VSD compared to controls [107]. Of these, *NOTCH1* [212], *HAND1* [213], *ZFPM2* [214], and *GATA3* [215], were predicted as targets of let-7e-5p, miR-222-3p and miR-433 [107].

In a microarray analysis of miRNAs in VSD and normal tissue samples, increased levels of *GJA1* and *SOX9* were associated with the decreased expression of miR-1-1 in VSD patients, and increased miR-181c expression was correlated with downregulated *BMPR2* levels, suggesting that both miRNAs are associated with the pathogenesis of VSD [105]. *GJA1* encodes the main cardiac gap junction channel that is responsible for the synchronous contraction of the ventricles [216]. *SOX9* plays an essential role in the pathway that controls the formation of the cardiac valves and septa, and the upregulation of the *SOX9* level was found to be associated with VSD in *Ets1*^−/−^ mice [217].

In a familial form of isolated ostium secundum ASD that affects four individuals in a family of five with autosomal dominant inheritance, whole genome sequencing identified a new mutation (c.1784T > C) in the 3′-untranslated region (3′UTR) of *ACTC1*, which encodes the predominant actin in the embryonic heart [109]. This mutation resulted in a new target site for miRNA-139-5p, a miRNA that is involved in cell migration, invasion, and proliferation [109]. In vitro analyses demonstrated functional interaction of miRNA-139-5p and *ACTC1* with the new mutation, providing evidence that a pathogenic mutation in the *ACTC1* 3′UTR that may be associated with familial isolated secundum ASD [109].

##### lncRNAs and CHD-

lncRNAs have diverse roles including transcriptional and post-transcriptional regulation in various biological processes, such as differentiation, cell proliferation and growth. Several lncRNAs have been implicated in cardiac development, including Alien [218], Braveheart [219,220], Carmen [221], Fendrr [222,223], Punisher [218], Terminator [218], and Upperhand [224]. Presently, there is limited evidence of the involvement of lncRNAs in CHDs. Several studies have demonstrated the importance of lncRNAs in septal defects. The lncRNA *SNHG6* has been shown to modulate the development of many human cancers [225,226,227,228]. From the perspective of cardiovascular development, *SNHG6* expression was decreased during embryonic heart development of mice, and in differentiation of P19 cells into cardiomyocytes. However, the expression of lncRNA *SNHG6* was observed to be increased in fetal cardiac tissues of VSD patients [229]. Experimental analyses demonstrated that in vitro overexpression of *SNHG6* inhibited P19 cell proliferation and induced apoptosis, as well as promoted cell differentiation into cardiomyocytes [229]. In addition, miR-101 was downregulated and Wnt/β-catenin pathway was activated, suggesting that *SNHG6* may contribute to VSD formation via negative regulation of miR-101 and activation of Wnt/β-catenin pathway [230,231].

Comparison of the transcriptomic profiles of cardiac tissues from VSD and normal hearts revealed upregulation of 880 lncRNAs, and downregulation of 628 lncRNAs in VSD. Of these, two lncRNAs, ENST00000513542 and RP11-473L15.2, were found to have an association with VSD [232]. More recently, association between lncRNA MALAT1 (metastasis-associated lung adenocarcinoma transcript 1) single nucleotide polymorphisms and occurrence of CHD, particularly to VSD and ASD, has been described in a Chinese population [233]. MALAT1 rs619586 GG allele was significantly associated with lower risk of CHD, and functional investigation indicated that G allele of rs619586 could trigger higher expression of MALAT1. This was a demonstration that the functional MALAT1 polymorphism rs619586 A > G was significantly associated with CHD susceptibility in Chinese population, potentially via regulating MALAT1 expression [233].

Despite the paucity of studies to elucidate the functional relevance of lncRNAs in the pathogenesis of CHD, lncRNAs may potentially be attractive clinical biomarkers of CHD. Specific lncRNAs aberrantly expressed in the plasma of pregnant women with typical fetal CHD may play a key role in the development of CHD. These differentially expressed lncRNAs (ENST00000436681, ENST00000422826, AA584040, AA709223 and BX478947) may have potential diagnostic value for prenatal diagnosis of fetal CHD, providing evidence that circulating plasma lncRNA may serve as novel biomarkers for CHD diagnosis [234].

##### circRNAs and CHD

Recently, a novel group of non-coding RNAs termed circular RNAs (circRNAs) that exist ubiquitously in the cytosol of eukaryotic cells was discovered [235,236,237]. Compared with linear RNAs, circRNAs uniquely undergo non-canonical splicing that circularized and lacks 3′ or 5′ end [238]. CircRNAs are largely proposed to function as miRNA sponges and were believed to antagonize miRNA-dependent gene regulation, thus contributing substantially to the competing endogenous RNA network [239]. Microarray expression analysis of plasma samples identified three circRNAs (hsa_circRNA_004183, hsa_circRNA_079265, and hsa_circRNA_105039) that were downregulated in plasma from children with CHD and may serve as novel non-invasive biomarkers for the diagnosis of CHD in children [240]. These findings were based upon a discovery cohort of 4 CHD and 4 matched healthy controls, and a subsequent validation cohort (40 CHD cases; 40 matched healthy controls) [240].

### 4.4. Other Epigenetic Mechanisms

#### 4.4.1. Sumoylation

Sumoylation is a post-translational modification in which small ubiquitin-related modifier (SUMO) proteins are covalently conjugated to target proteins via a series of enzymatic reactions. Evidence for sumolyation in CHD is largely limited to animal studies although its implication in human cardiovascular diseases (CVDs) has been emerging [241,242]. The protein SUMO1 is downregulated in failing hearts in humans and animals [243]. In mice both SUMO1^+/–^ and SUMO1^–/–^ exhibit congenital heart defects with high mortality, and these severe phenotype can be rescued by transduction of the *SUMO1* gene [244]. DNA sequence analysis from newborn screening blood spots revealed a single 16bp substitution in the SUMO1 regulatory promoter of a patient displaying both oral-facial clefts and ASDs [244]. In vitro study on this mutation demonstrated ~95% reduced activity of the *SUMO1* promoter suggesting a link between reduced sumoylation and CHDs [244].

#### 4.4.2. RNA Modifications

Increasing studies have identified several mRNA modifications that are responsible for various cardiac diseases. RNA methylation including N6-methyladenosine (m^6^A), N1-methyladenosine, 5–methylcytosine (m^5^C), N7-methylguanosine, N4-acetylcytidine, and 2′-O-methylation are novel epigenetic modifications that affect the homeostasis of cardiomyocytes. The role of RNA methylation in the pathophysiology of CVDs has been recently reviewed [245] with several reports suggesting a link between RNA modification and human CHD. While the role of *HNRNPA1* as an m^6^A reader is debatable [246], heterozygous mutations in *HNRNPA1* has been identified in subjects with CHD, and *Hnrnpa1* homozygous mutant mouse model exhibited complete penetrance of CHD [247]. NSUN2 is a well-known tRNA m^5^C modifying enzyme that reduces endonucleolytic tRNA cleavage [248], and altered *Nsun2* expression changes the total amount of m^5^C in the mRNA pool [249]. Whole exome sequencing of 17 families with diverse disorders revealed a mutation in *NSUN2* that resulted in congenital disorder (Noonan-like syndrome) that mainly affects the heart by causing hypertrophic cardiomyopathy, pulmonary valve stenosis, and ASDs [250].

Of all RNA modifications m^6^A is the most prevalent type and, to date, majority of the studies have focused on the mechanism of m^6^A methylation. Emerging and increasingly affordable high-throughput sequencing studies on m^6^A-SNP will further elucidate the mechanism underlying m^6^A-SNPs and CHDs and broaden our understanding between m^6^A-SNP and pathogenesis of CVDs. Likewise, a more comprehensive understanding of the biological function of RNA modifications might provide novel insights into the potential biomarkers and therapeutic approaches for CHD (and CVDs) in the future.

## 5. Current Challenges and Future Directions

Although animal models of the developing heart have facilitated important discoveries to improve our understanding of CHD etiology, a tractable human model system is still required to validate and build upon the findings from animal models. As epigenetic phenomenon is time-/stage- and tissue-specific, human heart tissues from developing embryos is largely inaccessible or limited for epigenetic molecular analysis. Therefore, in vitro systems may be powerful tools for studying early heart specification and cardiac differentiation. Whilst 2D monolayer approaches have successfully modeled early cardiac specification, they lack the ability to model morphogenetic events, and to recapitulate in vivo cardiac architecture and simulate cardiac tissue complexity and organization. To circumvent the intrinsic limitations of conventional 2D cell cultures, human cardiac organoids have been generated using 3D culture systems, which can more closely recapitulate endogenous cardiomyocyte tissues by incorporating key elements of the cardiac system, including supporting scaffolds and external stimuli [251]. In recent times, self-organizing mouse gastruloids have been used as a model to study the earliest stages of cardiogenesis with an in-vivo-like spatiotemporal fidelity [252]. Although gastruloids are no substitute for in vivo studies, they are still highly complex structure containing different cell types from all three germ layers, thereby conferring the feasibility to evaluate the dynamic interactions of endodermal and ectodermal tissues with cardiac progenitors as they undergo specification and maturation [252].

As evident from the curation of human studies in this review, bulk sequencing studies (e.g., transcriptomics) were performed on myocardial tissues procured from terminated foetuses or surgical samples or using blood samples. This is, in part, due to the inaccessibility to human heart tissues during the earliest stages of cardiogenesis, and the inability to detect the CHD until the second trimester when the heart is already fully formed. Studies using blood samples and/or bulk studies alone often overlook the spatiotemporal element of gene expression and its regulation, which is context-specific. The effective utilisation of scRNA-seq in in vivo animal models have facilitated the study of morphological sequelae enabling scientists to parse essential signalling and regulatory networks in cardiogenesis, uncovering stage-specific disturbances related to CHD [253]. Moving forward, we can look forward to optimisation of human in vitro models (e.g., gastruloids) to mimic the earliest stages of cardiogenesis and thereby facilitate the assessment of associated developmental defects. A greater diversity of well-established models in combination with advanced technologies (e.g., scRNA-seq, CRISPR gene editing) will allow us to address the outstanding conundrums of CHD.

## 6. Conclusions

It is evident that transcriptional regulation plays a crucial role for normal cardiovascular development and that, if deranged, CHD may be a consequence. In a majority of CHD cases, single gene mutation cannot explain a given patient’s CHD phenotype. It goes to suggest the role of additional mechanisms including transcriptional dysregulation and epigenetic mechanisms. Here we have summarised the roles of epigenetic regulation on cardiac development, and the clinical studies implicating the involvement of epigenetic dysfunction in CHD. In most of these studies, the functional contribution of individual epigenetic mechanism to CHD pathogenesis remains to be elucidated. Be that as it may, comparison between those differentially expressed genes or non-coding RNA identified in various CHD resulted in minimal overlap, suggesting that distinct molecular hallmark may apply to each CHD. Several studies in CHD patients were able to identify miRNA or lncRNA as biomarkers, supporting their use as predictive tools with potential diagnostic value for prenatal diagnosis of fetal CHD. With the rapid research advancement of epigenetics and developmental biology, more will be revealed on the etiology or origin of many CHDs. 

## Figures and Tables

**Table 1:** Genes and loci commonly associated with syndromic congenital heart disease.

Syndrome	Genes	Loci	Cardiac Disease	% CHD
Alagille	*JAG 1* *NOTCH2*	20p12.2 1p12-p11	PPS, TOF, PA	>90
CFC	*BRAF* *KRAS* *MAP2K1* *MAP2K2*	7q34 12p12.1 15q22.31 19p13.3	PVS, ASD, HCM	75
Cantu	*ABCC9*	12p12.1	PDA, BAV, HCM, CoA, PE, AS	75
Char	*TFAP2B*	6p12.3	PDA, VSD	58
CHARGE	*CHD7*	8q12	TOF, PDA, DORV, AVSD, VSD	75–85
Costello	*HRAS*	11p15.5	PVS, ASD, VSD, HCM, arrhythmias	44–52
DiGeorge	*TBX1*	22q11.2 deletion	Conotruncal defects, VSD, IAA, ASD, VR	74–85
Ellis-van Creveld	*EVC* *EVC2*	4p16.2 4p16.2	Common atrium	60
Holt-Oram	*TBX5*	12q24.1	VSD, ASD, AVSD, conduction defects	50
Kabuki	*KMT2D* *KDM6A*	12q13 Xp11.3	CoA, BAV, VSD, TOF, TGA, HLHS	50
Noonan	*PTPN11* *SOS1* *RAF1* *KRAS* *NRAS* *RIT1* *SHOC2* *SOS2* *BRAF*	12q24.13 2p22.1 3p25.2 12p12.1 1p13.2 1q22 10q25.2 14q21.3 7q34	Dysplastic PVS, ASD, TOF, AVSD, HCM, VSD, PDA	75
Williams-Beuren	7q11.23 deletion *(ELN)*	7q11.23	SVAS, PAS, VSD, ASD	80
Carpenter	*RAB23*	6p11.2	VSD, ASD, PDA, PS, TOF, TGA	50
Coffin-Siris	*ARID1B* *SMARCB1* *ARID1A* *SMARCB1* *SMARCA4* *SMARCE1*	6q25 22q11 1p36.1 22q11.23 19p13.2 17q21.2	ASD, AVSD, VSD, MR, PDA, PS, DEX, AS	20–44
Cornelia de Lange	*NIPBL* *SMC1L1* *SMC3*	5p13 Xp11.22 10q25	PVS, VSD, ASD, PDA	33
Mowat-Wilson	*ZEB2*	2q22.3	VSD, CoA, ASD, PDA, PAS	54
Rubinstein-Taybi	*CBP* *EP300*	16p13.3 22q13.2	PDA, VSD, ASD, HLHS, BAV	33
Smith-Lemli-Opitz	*DHCR7*	11q12-13	AVSD, HLHS, ASD, PDA, VSD	50

Abbreviations: AS, aortic stenosis; ASD, atrial septal defect; AVSD, atrioventricular septal defect; BAV, bicuspid aortic valve; CFC, cardiofaciocutaneous; CHARGE, coloboma, heart defects, choanal atresia, retarded growth and development, genital anomalies, and ear anomalies; CoA, coarctation of the aorta; DEX, dextrocardia; DORV, double-outlet right ventricle; HCM, hypertrophic cardiomyopathy; CHD, congenital heart disease; HLHS, hypoplastic left heart syndrome; IAA, interruption of aortic arch; MR, mitral regurgitation; PA, pulmonary atresia; PAS, pulmonary artery stenosis; PDA, patent ductus arteriosus; PE, pericardial effusion; PPS, peripheral pulmonary stenosis; PS, pulmonary stenosis; PVS, pulmonary valve stenosis; SVAS, supravalvular aortic stenosis; TGA, transposition of great arteries; TOF, tetralogy of Fallot; VR, vascular ring; and VSD, ventricular septal defect. *Note*: % CHD denotes the proportion of patients with the particular genetic syndrome affected by CHD. Adapted from [7].

**Table 2:** Cyanotic congenital heart diseases.

Cyanotic CHD	Brief Description
Tetralogy of Fallot (TOF)	A common cyanotic CHD; characterized by pulmonary stenosis/right ventricular outflow tract obstruction, VSD, over-riding aorta and hypertrophy of the right ventricle
Transposition of the great arteries (TGA)	Discordant ventriculoarterial connection—the right ventricle is connected to the aorta (instead of pulmonary artery), and left ventricle to pulmonary artery (instead of aorta)
Double outlet right ventricle (DORV)	Both the aorta and pulmonary artery arise predominantly or completely, from the right ventricle
Persistent truncus arteriosus	Failure of septation of the primitive truncus into the aorta and pulmonary artery, resulting in a single, common arterial trunk that overlies a large VSD
Hypoplastic left heart syndrome (HLHS)	Underdevelopment of the left-sided structures of the heart, including the ascending aorta, left ventricle and aortic and mitral valves

Note: TOF, TGA, DORV, and persistent truncus arteriosus are collectively known as conotruncal defects, as these lesions involve the conus and truncus arteriosus of the embryonic heart.

**Table 3:** DNA methylation in CHDs.

DNA Methylation					
Allele	Clinical Sample Size	Modification	Tissue Type	Cardiac Disease Phenotype	Reference
*NOX5*	21 VSD and 15 controls	Hypermethylation	Fetal myocardial tissue	VSD	[78]
*KIAA0310; RAB43; NDRG2*	21 VSD and 15 controls	Hypermethylation	Fetal myocardial tissue	VSD	[79]
*SIVA1*		Hypomethylation			
LINE-1*	32 TOF and 15 controls [80]; 48 TOF patients and 16 controls [81]	Hypomethylation	Right ventricular tissue samples [80]; Right ventricular outflow tracts [81]	TOF	[80]
*NKX2.5; HAND1; EGFR; EVC2; TBX5; CFC1B*	30 TOF and 6 controls [82]; 41 TOF and 6 control [83]	Hypermethylation	Right ventricular myocardium tissues	TOF, HLHS	[82,83]
*GATA4; MSX1*	6 Down syndrome with CHD, 6 Down syndrome without CHD, 6 isolated heart malformations, and 4 control	Hypermethylation	Whole heart tissue	AVSD, VSD, CoA, TOF, LHH, HAA, DORV, VSD, TOF, MVA, AVA, PFO, TVS; RHH, TA; ADA; TAV	[84]
*SCO2*	8 TOF, 8 ventricular septal defect, and 4 control	Hypermethylation	Myocardial biopsies	TOF, VSD	[85]
*ZFPM2*	43 TOF and 6 controls	Hypermethylation	Right ventricular outflow tract	TOF	[86]
*p16^INK4a^*	63 TOF and 75 controls	Hypermethylation	Whole blood	TOF	[87]
*BRG1*	24 CHD and 11 controls	Hypomethylation	Various cardiac tissues.	TOF, VSD, DCRV	[88]
*MTHFR*	40 Down syndrome without CHD; 40 mothers of Down syndrome with CHD, and 40 age matched control mothers	Hypermethylation	Whole blood	AVSD; VSD, ASD; TOF	[89]
*TBX20*	23 TOF and 5 controls [90]; 42 TOF and 6 controls [91]	Hypomethylation	Right ventricular myocardial tissues	TOF	[90]
*ZIC3*; *NR2F2*	Monozygotic twin pair discordant for DORV	Hypermethylation	Whole blood	DORV	[92]
*NRG1*	7 Down syndrome patients with CHD and 9 Down syndrome without CHD	Hypermethylation	Whole blood	Endocardial cushion-type	[93]

* Repetitive Long Interspersed Nucleotide Element-1. Cardiac disease abbreviations: ADA (Absent Ductus Arteriosus), ASD (Atrial Septal Defects), AVA (Aortic Valve Atresia), AVSD (Atrioventricular Septal Defect), CoA (Coarctation of the pre-ductal Aorta), DCRV (Double-Chambered Right Ventricle), DORV (Double Outlet Right Ventricle), HAA (Hypoplasia of the Ascending Aorta), HLHS (Hypoplastic Left Heart Syndrome), LHH (Left Heart Hypoplasia), MVA (Mitral Valve Atresia), PFO (Patent Foramen Ovale), RHH (Right Heart Hypoplasia), TA (Truncus Arteriosus), (Tetralogy of Fallot), TVS (Tricuspid Valve Stenosis), VSD (Ventricular Septal Defect).

**Table 4:** Histone modifications in CHDs.

Histone Modification					
Allele	Clinical Sample Size	Modification	Source Type	Cardiac Disease Phenotype	Reference
*WHSC1*	Case study	H3K36me3	In vivo mouse models [94]	HLH; WHS	[94,95,96]
*MLL2; CHD7; WDR5; KDM5A; KDM5B*	362 severe CHD cases and 264 controls	H3K4me	Whole blood	LVO; CTD	[97]
*UBE2B; RNF20; USP44*		H2BK120		CTD; HTX; LVO	
*SMAD2*		H3K27		HTX	
*EBAF*	16 VSD and 16 normal fetuses at 22–28 weeks of gestation.	H4ac	Myocardial tissue	VSD	[98]
*RNF20; RNF40; UBE2B*	2645 case trios and 1789 control trios.	H2Bub1	Whole blood or sputum	Dextrocardia; RAI; TAPVR; CAVC; PA; L-TGA; HLHS; TOF, RAA	[99]
*JMJD1C; RREB1; MINA; KDM7A*	89 severe CHD cases and 95 controls	H3K27/H3K9	Whole blood	CTD	[100]
*KAT2B*	400 Chinese Han	HAT	Whole blood	TOF, TA and TGA, VSD, AVSD and PDA	[101]
*PRDM6*	35 individuals and their extended kindreds	H3K9me2/H4K20me2	Whole blood	N-PDA	[102]
*KANSL1*	253 diseased patients	H4K16ac	Whole blood	TOF	[103]

Cardiac disease abbreviations: AVSD (Atrioventricular Septal Defect), CAVC (Complete Atrioventricular Canal), CTD (Conotruncal Defects), HLH (Hypoplastic left heart), HLHS (Hypoplastic Left Heart Syndrome), HTX (Heterotaxy), L-TGA (Levo-Transposition of The Great Arteries), LVO (Left Ventricular Obstruction), N-PDA (Nonsyndromic Patent Ductus Arteriosus), PA (Pulmonary Atresia), PDA (Patent Ductus Arteriosus), RAA (Right Aortic Arch), RAI (Right Atrial Isomerism), TA (Truncus Arteriosus), TAPVR (Total Anomalous Pulmonary Venous Return), TGA (Transposition of the Great Arteries), TOF (Tetralogy of Fallot), VSD (Ventricular Septal Defect), WHS (Wolf-Hirschhorn syndrome).

**Table 5:** Non-coding RNA in CHDs.

Non-Coding RNA					
Allele	Clinical Sample Size	Modification	Tissue Type	Cardiac Disease Phenotype	Reference
miR-196a (rs11614913 CC)	1324 CHD and 1783 controls	Increased mature miR-196a expression	Whole blood	TOF, VSD; ASD	[104]
miR-1-1	28 VSD and 9 controls	Upregulates *GJA1* and *SOX9*	Heart tissue	VSD	[105]
miR-181c		Downregulates *BMPR2*		VSD	
miR-1, miR-206	30 TOF and 10 controls	Upregulates Cx43	Myocardium tissue	TOF	[106]
let-7e-5p; miR-222-3p; miR-433	3 VSD and 3 controls	Downregulated	Blood plasma	VSD	[107]
miR-184	10 CHD and 10 controls	Downregulated	Right ventricular outflow tract	Cyanotic cardiac defects	[108]
miRNA-139-5p	5 family individuals	(c.1784T > C) gain-of-function	Whole blood	ASDII	[109]
miR-518e, miR-518f, and miR-528a	7 Down syndrome patients with AVSD and 22 Down syndrome patients without CHD	Downregulates *AUTS2*	Down syndrome lymphoblastoid cell lines (GSE34457)	AVSD	[110]
miR-518a, miR-518e, miR-518f, and miR-96		Downregulates *KIAA2022*			
miR-138 (rs139365823)	857 CHD and 938 controls	Upregulates miR-138	Whole blood	VSD; ASD; TOF; PDA	[111]

Cardiac disease abbreviations: ASD (Atrial Septal Defects), ASDII (Isolated ostium secundum atrial septal defect), AVSD (Atrioventricular Septal Defect), PDA (Patent Ductus Arteriosus), TOF (Tetralogy of Fallot), VSD (Ventricular Septal Defect).
